# Latent class analysis of acceptability and willingness to pay for self-HIV testing in a United States urban neighbourhood with high rates of HIV infection

**DOI:** 10.7448/IAS.20.1.21290

**Published:** 2017-01-17

**Authors:** Amy Nunn, Lauren Brinkley-Rubinstein, Jennifer Rose, Kenneth Mayer, Thomas Stopka, Caitlin Towey, Julia Harvey, Karina Santamaria, Kelly Sabatino, Stacey Trooskin, Philip A Chan

**Affiliations:** ^a^ Department of Behavioral and Social Sciences, Rhode Island Public Health Institute, Providence, RI, USA; ^b^ School of Public Health, Brown University, Providence, RI, USA; ^c^ Department of Social Medicine, University of North Carolina, Chapel Hill, NC, USA; ^d^ Center for Health Equity Research, University of North Carolina, Chapel Hill, NC, USA; ^e^ Quantitative Statistical Center, Wesleyan University, Middletown, CT, USA; ^f^ Fenway Health, Harvard Medical School, Beth Israel Deaconess Medical Center, Boston, MA, USA; ^g^ School of Medicine, Tufts University, Boston, MA, USA; ^h^ College of Medicine, Drexel University College of Medicine, Philadelphia, PA, USA; ^i^ Division of Infectious Diseases, Warren Alpert Medical School of Brown University, Providence, RI, USA; ^j^ Division of Infectious Diseases, The Miriam Hospital, Providence, RI, USA

**Keywords:** HIV self-test, geography, home-based testing, neighbourhood

## Abstract

**Introduction**: Acceptability and willingness to both take and pay for HIV self-tests (HIVSTs) in US neighbourhoods with high rates of HIV infection are not well understood.

**Methods**: We surveyed 1,535 individuals about acceptability and willingness to take and pay for an HIVST in a predominately African American neighbourhood with 3% HIV seroprevalence. We recruited individuals presenting for HIV screening services in a community-based programme. Latent class analysis (LCA) grouped individuals with similar patterns of HIV-risk behaviours and determined which groups would be most willing to use and buy HIVSTs.

**Results**: Nearly 90% of respondents were willing to use an HIVST; 55% were willing to buy HIVSTs, but only 23% were willing to pay the market price of US $40. Four distinct groups emerged and were characterized by risk behaviours: (1) low risk (*N* = 324); (2) concurrent partnerships (*N* = 346); (3) incarceration and substance use (*N* = 293); and (4) condomless sex/multiple partners (*N* = 538). Individuals in the low-risk class were less willing to self-test compared to concurrent sexual partners (OR = 0.39, *p* = .003) and incarceration and substance use (OR = 0.46, *p* = .011) classes. There were no significant differences across classes in the amount individuals were willing to pay for an HIVST.

**Conclusions**: HIVSTs were overwhelmingly acceptable but cost prohibitive; most participants were unwilling to pay the market rate of US $40. Subsidizing and implementing HIVST programmes in communities with high rates of infection present a public health opportunity, particularly among individuals reporting condomless sex with multiple partners, concurrent sexual partnerships and those with incarceration and substance use histories.

## Introduction

Approximately 1.2 million Americans are living with HIV [1,2]. HIV testing is a critical gateway to HIV/AIDS treatment and care. Nationwide, 13% of HIV-infected people are unaware of their infection [3]. Individuals unaware of their HIV infection contribute to nearly 50% of new HIV transmissions in the United States (US) [4]. Since 2006, the Centers for Disease Control and Prevention (CDC) has recommended that all adults between the ages of 13 and 65 be tested for HIV at least once annually, and that HIV screening be conducted routinely in clinical settings [5]. However, only 54% of Americans have ever undergone HIV screening, and only 22% of all Americans have been tested in the past year [6].

Many individuals at high risk for contracting HIV still do not have access to clinical HIV screening services [7,8]. Racial and ethnic minorities and individuals from lower socio-economic strata are less likely to be aware of their HIV infection [9,10]. African Americans and Hispanics/Latinos have eight and three times the infection rates of Whites [11], are more likely to present for care late in the course of their infection [12], and have poorer outcomes at every point along the HIV care continuum [13,14]. Additionally, African Americans and Hispanics are more likely to live in urban neighbourhoods with high levels of concentrated poverty and structural and social barriers that may prohibit access to healthcare services, and HIV testing and care [15].

One potential opportunity to expand HIV screening to populations with limited access to HIV screening and care services, and particularly urban communities of colour, is through HIV self-tests (HIVSTs) [9,16,17]. In July 2012, the US Food and Drug Administration approved the “OraQuick® In-Home HIV Test” – the first over-the-counter, HIVST [18]. The HIVST retails for approximately US $40 in pharmacies and provides results in 20 minutes; individuals with reactive results are instructed to see a medical professional for confirmatory testing and care.

HIVSTs provide an opportunity to expand HIV testing in urban communities. Many groups with high HIV infection rates have limited healthcare infrastructure; therefore, HIVSTs might fill unmet needs for screening [9,19]. Moreover, HIVSTs may be particularly important in communities of colour with high rates of stigma; HIVSTs can also provide opportunities for individuals to self-test who would prefer not to test in clinical settings [20–22]. While HIVSTs have the potential to help reduce disparities in HIV screening, treatment and care, HIVST acceptability has not been well explored in urban communities of colour with high HIV infection rates.

Little is known about acceptability or affordability of HIVSTs, particularly in the most heavily impacted urban neighbourhoods in the US and among individuals at highest risk for acquiring HIV [9]. One recent study found that knowledge related to HIV transmission, treatment and concern about HIV in an individual’s community was associated with willingness to use an HIVST [23]. Another study suggested the retail price of the HIVSTs was too high – with most people preferring a price closer to US $22 [24]. In addition, a study among transwomen in San Francisco found that most were willing to use HIVSTs, but that price was a critical determinant of whether or not they would actually use them. Participants relayed that cost was the biggest barrier to using HIVSTs but that they would be willing to pay as much as $US $20 [25].

The current study is based on a geographically-focused, community-based, HIV and hepatitis C (HCV) testing, treatment and retention-in-care implementation research programme in a predominately African American neighbourhood in Philadelphia, Pennsylvania (PA) with 2–3% HIV seroprevalence and limited access to HIV screening and care services [26]. In an effort to expand HIV screening and care services in this urban community, this programme combined social marketing and community mobilization, door-to-door HIV and HCV outreach, and screening in non-clinical and clinical settings; the results of the screening and linkage to care programme have been presented elsewhere [17,27,28]. Among participants in the programme, we explored acceptability, affordability and attitudes about purchasing HIVSTs among participants.

## Methods

We collected information about attitudes related to HIVSTs as well as willingness to pay for HIVSTs among individuals who presented for HIV and HCV screening services on a mobile medical unit in urban Philadelphia between December 2012 and January 2014. Trained HIV/HCV testers conducted surveys face-to-face in a private room. Survey answers were recorded on encrypted tablets using *Illume* ^TM^ software (*Datstat*, Washington). Participants did not receive monetary compensation, but received free HIV and HCV testing, linkage to care services and HIV and HCV treatment when necessary. All participants provided informed consent. The Miriam Hospital institutional review board approved this study.

The survey included questions about demographic, behavioural, structural, and social factors, including information on sexual partners and risk behaviours, and HIV and HCV testing history. Specific questions about HIVSTs explored whether participants would use an HIVST, whether they thought that their friends and family would use a self-test, how much participants would be willing to pay for an HIVST, and whether they would go to a doctor if the HIVST result were reactive.

## Measures

Participant demographic covariates included age (separated into four ordinal age categories: twenty years old or younger, 21–30, 31–39 and 40 or older), sex, education (less than a high school degree, high school degree and at least some college education), sexual orientation (straight, gay/lesbian or bisexual), how often the participant attended religious services (never, sometimes, at least once a week), employment status (full/part time or unemployed), and whether or not the participant had medical insurance or a primary care physician.

A total of 12 variables (hereafter called classification variables) were included in the latent class analysis (LCA) to identify latent classes of individuals. These classification variables were selected to represent a range of established HIV risk factors in order to identify comprehensive HIV-risk profiles. They included measures of incarceration history, perceived HIV risk, prior HIV testing, sexual behaviour and substance use. The survey assessed *incarceration history* by asking participants whether they had ever been incarcerated (yes or no). *Perceived HIV risk* was assessed by asking participants to rate their risk for HIV on a four-point Likert scale ranging from “not at risk” to “high risk”. Responses on this variable were dichotomized into “moderate to high risk” and “low to no risk”. Participants were also asked whether they had ever been screened for HIV (yes or no).

*Sexual behaviour* was assessed by asking participants whether they had engaged in condomless sex (anal, vaginal, oral) within the past year (yes or no); whether, in the past year, they had engaged in condomless sex in exchange for drugs, alcohol, gifts, food, or shelter; and if they had ever had sex with someone who was HIV positive or someone with a history of injection drug use. Participants who responded yes to at least one of these questions were coded as having engaged in higher-risk sexual encounters. Participants were also asked whether they had engaged in concurrent sexual partnerships, defined as whether they were engaged in sexual partnerships that overlapped in time (yes or no). They were also asked the number of partners they had in the past year (0–1, 2–5, 6 or more) and whether they believed their most recent partner had concurrent sexual partners during the time period they were also in a sexual partnership (yes or no).

*Substance use* was measured with a number of questions related to drug and alcohol use. Questions included: how often have you had six or more drinks on one occasion (coded as less than monthly vs. monthly or more), and binary variables asking did you drink alcohol the last time you had sex (yes or no), have you ever used crack, cocaine, heroin, prescription drugs without a prescription, ecstasy, special K or crystal methamphetamine (yes or no).

*HIVST outcomes* were assessed by asking questions related to participants’ attitudes about and willingness to buy HIVSTs. To assess attitudes, two questions were asked: (1) how likely are you to take an HIVST if it was provided for free, and (2) how likely are you to buy an HIVST. Both questions originally had a five-point Likert response scale (very unlikely, unlikely, neutral, likely and very likely) but were transformed into a binary variable for analysis – “likely”, which included likely and very likely, vs. “not likely”, which included “neutral”, “unlikely” and “very unlikely”. To assess willingness to buy an HIVST, participants were asked how much they would be willing to pay for an HIVST at their local pharmacy. Response options included “not interested in buying a test”, $0–10, $11–20, $21–30, $31–40, or $40 or more. A binary variable was created in which all price variables other than $40 or more were combined and coded as 0, and $40 or more was coded as 1 to understand whether participants would be willing to pay $40 or more (the approximate retail value of an HIVST) or less.

## Data analysis

LCA was used to identify classes based on perceived HIV risk, HIVST history, past incarceration, sexual behaviour and substance-use variables. Classes are considered latent because class membership is not directly observed; rather it is inferred (probabilistically) based on an individual’s pattern of responses across a set of variables (i.e. their response profile). Unlike traditional regression analyses, LCA provides a multidimensional perspective that shows how variables work together to predict multiple HIVST outcomes such as HIVST uptake, likeliness to pay, and payment thresholds. Using LCA to discover underlying groups of individuals with similar risk response profiles allowed us to identify which kinds of group profiles would be most likely to both use and pay for HIVSTs. This method differs from multivariate analyses that examine the independent association of each variable with the response variable while holding other variables constant at a certain value. In other words, by identifying groups of individuals that share similar response profiles on the 12 classification variables, LCA provides information on how multiple variables interact with each other to predict response variables. This differs from traditional regression methods that would require a large number of interaction terms, which in turn, can make a regression model unwieldy and challenging to interpret.

A series of LCA models specifying 1–6 latent classes were tested using the Mplus software package [29]. To avoid the likelihood of converging on a local maximum, 500 start values were generated for each model. Indices used to determine the optimal LCA solution included the sample size-adjusted Bayesian Information Criterion (BIC) and the adjusted Lo Mendell Rubin (LMR) likelihood ratio test for model fit which tests the null hypothesis of no improvement in fit for the model under consideration compared to a model with one less class. Entropy, which measures the extent to which classes are distinct from one another, the average posterior probability of class membership, and the interpretability of the classes, was also considered. Logistic regression (for binary classification variables) and multinomial logistic regression (for categorical classification variables) were used to test for significant differences between classes on the latent class classification variables.

After identifying the LCA model with the number of classes that had the optimal LCA solution based on the criteria above, we added the participant demographic characteristics (age, gender, education, sexual orientation, religious services attendance, employment, medical insurance and primary care physician) to this model as covariates. In addition, we included the two outcome variables measuring likelihood of taking and buying an HIVST. We did not include amount willing to pay for an HIVST because it represented only the portion of the sample that indicated interest in buying an HIVST. We did not include race as a covariate because 89% of the sample was African American. However, we conducted a secondary multinomial logistic regression analysis, adjusting for the covariates, to determine whether the classes differed in the amount willing to pay.

Covariate prevalence rates and 95% confidence intervals were calculated for each latent class, and latent classes were compared on the outcomes using multinomial logistic regression analysis. Unlike traditional “classify then analyze” approaches that assume perfect classification, this approach provides a test of statistical significance for differences between classes on the outcomes that takes into account the posterior probabilities of class membership, thus avoiding the assumption of perfect classification.

## Results

**Figure 1.**
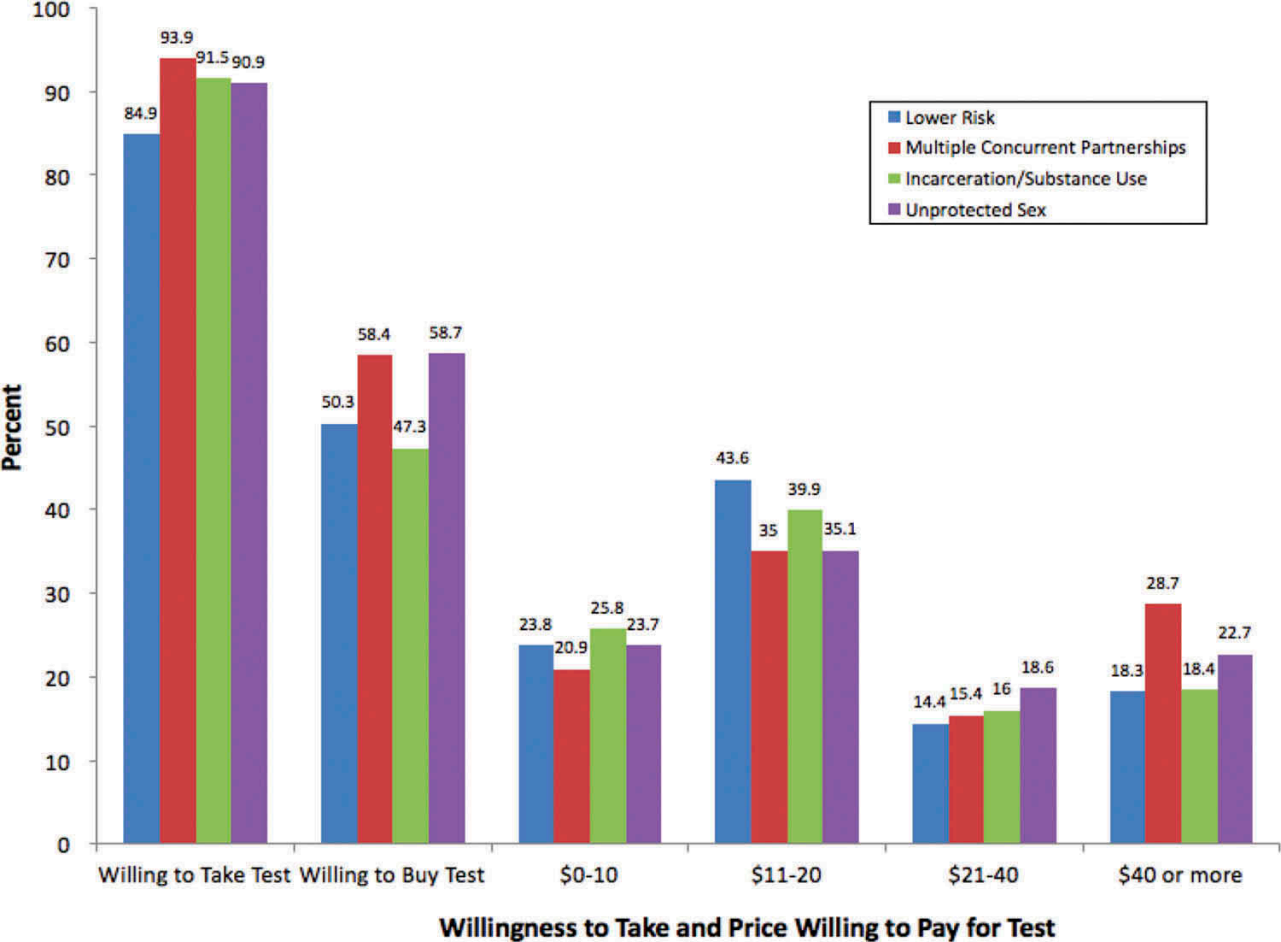
Willingness to take HIVST and maximum price willing to pay for HIVST by latent class.

LCA was conducted on participants with complete data on demographic covariates (1,501 of the 1,535 participants; 97%). The sample was 50% female (*n* = 751) and the majority (90%) was African American (*n* = 1362) and single (79%, *n* = 1198). Nearly half (46%, *n* = 597) were between the ages 13–20 (Table 1). Figure 1 highlights willingness to take and buy HIVSTs, as well as the maximum price participants were willing to pay for the HIVST. The majority (90%, *n* = 1357) of the sample indicated that they would be likely or very likely to take a free HIVST if it were provided and 55% (*n* = 819) would be likely or very likely to buy an HIVST. However, among those willing to test, only 23% (*n* = 228) of the sample indicated that they would be willing to pay the estimated retail price of $40 for the test. Nearly all (97%) reported they would be likely or very likely to seek medical care if their test results were reactive.

**Table 1.** Sample demographic characteristics (*N* = 1,535)

Variable	*N* (%)
Female	751 (49.5)
African American	1362 (89.8)
Single	1198 (79.0)
Age	
*13–20*	597 (45.5)
*21–29*	224 (17.1)
*30–39*	241 (18.4)
*40 or older*	249 (19.0)
Education	
*Less than high school*	260 (17.1)
*High school degree*	765 (50.4)
*At least some college education*	492 (32.4)
Sexual orientation	
*Heterosexual*	1357 (89.9)
*Same-sex*	64 (4.2)
*Bisexual*	88 (5.8)
Employed at least part time	712 (47.1)
Religious services attendance	
*Never*	945 (62.3)
*Sometimes*	265 (17.5)
*At least weekly*	306 (20.2)
Has health insurance	932 (61.4)
Has a primary care physician	920 (60.7)

A comparison of LCA model fit indices based on latent class models with no covariates showed that a 4-class solution was preferred (Table 2). The 4-class solution provided the lowest log likelihood and sample size adjusted BIC, and entropy was good at 0.74. In addition, the LMR test indicated a significant improvement in fit over a 3-class model. Although the LMR suggested a significant improvement in fit for the 5-class model over the 4-class model (*p* = 0.046), the log likelihood and BIC values were higher for the 5-class model, which was indicative of poorer fit for the 5-class model. Consequently, we chose to interpret the more parsimonious 4-class model. The average probability of latent class membership was 0.87 for Class 1, 0.88 for Class 2, 0.89 for Class 3 and 0.88 for Class 4, providing additional support that the classes were distinct.

**Table 2.** Latent class analysis fit indices for latent class models and class membership probabilities for 4 class model with covariates and home HIV test outcomes

Model	Log likelihood (# of parameters)	Sample size adjusted BIC	Lo-Mendell-Rubin p-value for k-1 classes	Entropy
1 class	−10053 (14)	20163	NA	NA
2 classes	−9424 (29)	18968	0.000	0.70
3 classes	−9269 (44)	18720	0.188	0.75
4 classes	−9155 (59)	18555	0.008	0.74
5 classes	−9225 (74)	18756	0.046	0.76
6 classes	−9207 (89)	18781	0.746	0.71
4 classes with covariates and self-HIV test outcomes	−8874 (95)	18142	0.002	0.78
Most Likely Latent Class Membership Probabilities				
	Class 1	Class 2	Class 3	Class 4
Class 1	**0.87**	0.01	0.03	0.08
Class 2	0.01	**0.88**	0.04	0.08
Class 3	0.05	0.04	**0.89**	0.02
Class 4	0.05	0.07	0.01	**0.88**

The 4-class model was then tested including the covariates and the two HIVST outcomes. The BIC for this model was lower than it was for the 4-class model with no covariates, suggesting an improvement in fit for the models with the covariates and the outcomes. Entropy and average latent class membership probabilities were improved as well (Table 2). Estimated prevalence rates and confidence intervals for the variables used to identify latent classes are shown in Table 3.

**Table 3.** Estimated percentages with 95% confidence intervals for LCA classification variables

	Lower risk (*N* = 324; 21.6%)	Concurrent partnerships (*N* = 346; 23.0%)	Incarceration/substance use (*N* = 293; 19.5%)	Condomless sex/multiple partners (*N* = 538; 35.8%)
Variable	% (95% CI)	% (95% CI)	% (95% CI)	% (95% CI)
Perceived moderate or high risk for HIV	**7.9** (2.7, 13.1)	31.8 (25.7, 37.8)	34.4 (27.1, 41.7)	20.8 (16.9, 24.7)
Ever tested for HIV	**74.5 (**68.4, 80.5)	91.0 (87.3, 94.7)	**93.4** (90.1, 96.8)	**84.8** (81.4, 88.3)
Ever incarcerated	15.2 (5.2, 25.2)	50.8 (43.3, 58.3)	**77.4** (70.1, 83.8)	11.2 (7.0, 15.4)
*Sexual behaviour*				
Engaged in condomless sex in past year	**41.8** (32.1, 51.4)	**88.2** (84.0, 92.5)	76.1 (69.9, 82.3)	**78.7** (74.4, 83.0)
Engaged in risky sex ever*	5.0 (1.8, 8.3)	7.1 (3.7, 10.4)	**35.8** (27.5, 44.1)	3.6 (1.5, 5.8)
Number of partners past year				
0–1	**96.4** (91.4, 100)	5.1 (0, 10.0)	47.2 (37.3, 57.2)	**69.7** (63.0, 76.4)
2–5	3.4 (0, 8.3)	**62.0** (55.4, 68.5)	37.1 (29.8, 44.4)	**30.0** (23.9, 36.1)
6 or more	<1.0 (0, 1.0)	**32.9** (25.6, 40.2)	15.7 (9.8, 21.5)	<1.0 (0, 1.8)
Participant had other partners during most recent sexual relationship	3.3 (0, 6.1)	**61.0** (51.6, 70.3)	38.4 (29.4, 47.4)	2.9 (0, 5.7)
Partner had other partners during participant’s most recent sexual relationship				
No	70.9 (63.8, 78.0)	27.3 (20.1, 34.5)	38.6 (30.6, 46.6)	63.4 (58.1, 68.7)
Yes	**8.4** (4.3, 12.5)	**39.1** (31.9, 36.3)	31.8 (23.8, 39.8)	13.0 (9.3, 16.7)
Don’t know	20.7 (14.3, 27.0)	**33.6** (27.8, 39.4)	29.6 (23.6, 35.6)	23.6 (19.3, 28.0)
*Alcohol and other drug use*				
Binge drinking	3.1 (0, 7.3)	14.8 (9.7, 19.99)	**28.8** (22.5, 53.2)	4.4 (2.1, 6.6)
Participant used alcohol at last sex	9.5 (2.3, 16.7)	21.8 (16.0, 27.6)	**45.5** (38.4, 52.6)	6.6 (3.6, 9.6)
Cocaine/crack use ever	11.7 (5.1,18.3)	<1.0 (0, 2.6)	**74.9** (62.8, 87.0)	0.0 (0,0)
Other drug use ever	2.4 (0, 5.5)	15.5 (10.8, 20.1)	**43.1** (34.2, 51.7)	3.0 (1.0, 4.9)

Note: * includes sex with some who was HIV positive, sex with someone who had an history of being an injection drug user, and sex in exchange for things like drugs, alcohol, gifts, food, or shelter. Values in bold are defining characteristics of the latent classes. Dashes indicate that the number endorsing that item was too small in one or both classes to get a reasonable parameter estimate.

## Class 1: lower risk

Class 1 (*N* = 324, 21.6% of the entire sample) had the lowest likelihood of having been tested for HIV among the four classes. This class was least likely to perceive any risk for acquiring HIV, with only 8% indicating they believed they were at risk for HIV. Class 1 also had the fewest number of recent sexual partners, with 96% indicating that they had 0–1 partners in the past year. This class also had the lowest likelihood of engaging in condomless sex (42%), the lowest rate of participant (3%) and partner (8%) concurrency, and relatively low rates of alcohol and other drug use, ranging 2% for other drug use to 9.5% for using alcohol at last sex. This class was labelled “Lower Risk”.

## Class 2: concurrent sexual partnerships

Participants in Class 2 (*N* = 346, 23.0% of the entire sample) were most likely to report engaging in condomless sex (88%) and had the highest rate of participant (61%) and partner (39%) concurrency. Although less likely to have a history of incarceration compared to the third class, an estimated 51% of participants in this class reported incarceration histories; 22% reported using alcohol at last sex, and 15% reported binge drinking and other drug use. This class was labelled “Concurrent Sexual Partnerships”.

## Class 3: incarceration and substance use

Class 3 participants (*N* = 293, 19.5% of the entire sample) were the most likely to report having engaged in a higher-risk sexual encounter (36%) and were most likely to have been incarcerated (77%). Participants in this class had the highest rate of alcohol and other drug use. An estimated 29% reported binge drinking, 45% used alcohol at last sex, 75% used cocaine and/or crack, and 43% reported other drug use. Participants in this class were more likely than Lower Risk and Condomless Sex/Multiple Partners class participants to believe they were at risk for HIV (34%) and to have been tested for HIV (93%). This class was labelled “Incarceration and Substance Use”.

## Class 4: condomless sex/multiple partners

Finally, the fourth and largest class (*N* = 538, 35.8% of the entire sample) was somewhat similar to the Lower-Risk class, with the exception that they were more likely to engage in condomless sex (79%), more likely to report having had 2–5 partners in the past year (30%), more likely to believe they were at moderate-to-high risk of contracting HIV (21%), and more likely to report having been tested for HIV in the past (85%). This class was labelled “Condomless Sex/Multiple Partners”.

## Latent class differences in demographic covariates

Table 4 shows prevalence rates in percentages for the four classes on the demographic covariates, with 95% confidence intervals. All participants in the Lower-Risk class were at least thirty years old, with the majority (96.6%) at least forty years old, which was significantly older than the other three classes as evidenced by non-overlapping confidence intervals. Compared to the other three classes, the Lower-Risk class also had a significantly greater percentage of heterosexual participants (96.3%), participants attending religious services at least weekly (33.3%), and had the lowest percentage of participants who were currently single (54.9%). The Concurrent Partnerships class had significantly fewer women (21.1%), and significantly fewer participants with health insurance (49.4%) or a primary care physician (45.1%). Compared to the other three classes, the Incarceration and Substance Use class had a significantly lower percentage of participants who were employed full time (29.4%), and included a high percentage of men were. Finally, the Condomless Sex/Multiple Partners class had a significantly greater percentage of women (70.8%) and had high percentages of individuals who had attended some college, had health insurance, and a primary care physician. In addition, this group had a significantly higher percentage of participants between the age of 13–20 (22.3%), and have participants who were employed at least part time (57.6%) compared to the other three classes.

**Table 4.** Estimated prevalence rates and 95% confidence intervals for demographic covariates

Variable	Lower risk% (95% CI)	Concurrent partnerships % (95% CI)	Incarceration and substance use % (95% CI)	Condomless sex/multiple partners % (95% CI)
Female	59.0 (53.4, 64.4)	**21.1 (16.9, 25.8)**	34.1 (28.7, 39.9)	**70.8 (66.8, 74.6)**
Age				
*13–20*	0 (0.0, 0.0)	14.5 (10.9, 18.7)	0 (0.0, 0.0)	**22.3 (18.9, 26.1)**
*21–29*	0 (0.0, 0.0)	48.3 (42.9, 53.7)	8.2 (5.3, 11.9)	45.9 (41.6, 50.2)
*30–39*	3.4 (1.7, 6.0)	22.3 (18.0, 27.0)	16.7 (12.6, 21.5)	23.2 (19.7, 27.0)
*40 or older*	**96.6 (94.0, 98.3)**	15.0 (11.4, 19.2)	75.1 (69.7, 79.9)	8.6 (6.3, 11.2)
Education				
*Less than high school*	14.2 (10.6, 18.5)	20.2 (16.1, 24.9)	29.4 (24.2, 34.9)	10.4 (7.6, 12.9)
*High school degree*	46.9 (50.7, 51.6)	58.7 (53.3, 63.9)	50.8 (45.0, 56.7)	48.8 (43.5, 52.1)
*At least some college*	39.8 (34.5, 45.4)	21.1 (16.9, 25.8)	19.8 (15.4, 24.8)	42.2 (38.0, 46.5)
Sexual orientation				
*Heterosexual*	96.3 (93.6, 98.1)	88.4 (84.6, 91.6)	84.3 (79.6, 88.3)	90.3 (87.5, 92.7)
*Same-sex*	2.2 (0.9, 4.4)	4.1 (2.2, 6.8)	6.8 (4.2, 10.4)	4.3 (2.7, 6.4)
*Bisexual*	1.5 (0.5, 3.6)	7.5 (5.0, 10.8)	8.9 (6.8, 12.7)	5.4 (3.6, 7.7)
Employed at least part time	46.0 (40.5, 51.6)	46.2 (40.9, 51.7)	**29.4 (24.2, 34.9)**	**57.6 (53.3, 61.8)**
Currently single	**54.9 (49.3, 60.4)**	90.1 (87.2, 93.6)	75.1 (69.7, 79.9)	88.3 (85.3, 90.1)
Attends religious services				
*Never*	46.6 (41.1, 52.2)	69.7 (64.5, 74.5)	63.1 (57.3, 68.7)	66.5 (62.4, 70.5)
*Sometimes*	20.1 (15.8, 24.8)	15.9 (12.2, 20.2)	14.7 (10.8, 19.3)	19.0 (15.7, 22.5)
*At least weekly*	**33.3 (28.2, 38.8)**	14.5 (10.9, 18.6)	22.2 (17.6, 27.4)	14.5 (11.6, 17.8)
Has health insurance	67.6 (62.2, 72.3)	**49.4 (44.0, 54.8)**	62.1 (56.3, 67.8)	64.9 (60.7, 68.9)
Has a primary care physician	67.9 (62.5, 73.0)	**45.1 (39.8, 50.5)**	59.4 (53.5, 65.1)	66.9 (62.8, 70.1)

Note: Values in bold indicate significantly different percentages for each class compared to the other three classes as evidenced by non-overlapping 95% confidence intervals.

## Latent class differences related to HIVST variables

Figure 1 shows the unadjusted percentage of participants in each class for the three HIVST outcomes. The Concurrent Sexual Partnerships class had the highest percentage of participants who indicated they would be likely to take an HIVST if it were free (94%), followed by the Incarceration and Substance Use (92%) and Condomless Sex/Multiple Partners (90%) classes. The Lower-Risk class had the lowest percentage of participants who indicated that they would likely take a free HIVST (84.9%), which was significantly lower compared to the Concurrent Sexual Partnerships class (OR = 0.39, *p* = 0.003) and the Incarceration and Substance Use class (OR = 0.46, *p* = 0.011) (see Table 5).

**Table 5.** Odds ratios and 95% confidence intervals for differences between latent classes in HIVST outcomes

Variable	Lower risk vs. concurrent partnerships OR (95%CI)	Lower risk vs. incarceration/substance use OR (95%CI)	Lower risk vs. condomless sex/multiple partners OR (95%CI)	Concurrent partnerships vs. condomless sex/multiple partners OR (95% CI)	Incarceration/substance use vs. concurrent partnerships OR (95%CI)	Incarceration/substance use vs. condomless sex/multiple partners OR (95%CI)
Likely to take a free HIVST	**0.39 (0.21, 0.73)**	**0.46 (0.25, 0.84)**	0.63 (0.37, 1.07)	0.62 (0.34, 1.14)	0.86 (0.44, 1.69)	0.68 (0.39, 1.20)
Likely to buy HIVST	0.86 (0.60, 1.24)	1.30 (0.89, 1.96)	0.78 (0.55, 1.09)	1.11 (0.81, 1.53)	**0.66 (0.46, 0.95)**	**0.60 (0.43, 0.82)**

Note: Values in bold indicate significant differences between latent classes.

Among participants who indicated they would take a free HIVST, 58% of members of the Concurrent Sexual Partnerships and Condomless Sex/Multiple Partners classes indicated they would be likely to *buy* an HIVST, followed by 50% of the Lower-Risk class and 47% of the Incarceration and Substance Use classes. The Incarceration and Substance Use class had a significantly lower proportion of participants who would be willing to buy a home test compared to the Concurrent Sexual Partnerships class (OR = 0.66, *p* = 0.025) and the Condomless Sex/Multiple Partners class (OR = 0.60, *p* = 0.002) (Table 5).

The majority of participants in all classes were willing to pay $11–20 for an HIVST kit (Figure 1). There were no significant differences between the classes in the amount participants were willing to pay.

## Discussion

HIVST was highly acceptable among participants in this study. Participants were willing to take HIVSTs in an urban, mostly African American US neighbourhood with high rates of HIV infection and limited access to clinical HIV screening and care services. Participants overwhelmingly reported that they would be willing to take an HIVST, and that their friends, family and loved ones would also be willing to self-test. In addition, LCA revealed four distinct groups representing (1) lower-risk participants, (2) participants with high rates of concurrent sexual partnerships, (3) participants with high rates of incarceration and substance use, and (4) participants reporting condomless sex and multiple sexual partnerships.

These classes had varying degrees of willingness to undergo HIV self-testing. Compared to the other classes, the Lower-Risk class was less willing to take a free HIVST. The incarceration and substance use class was no less likely than the Concurrent Sexual Partners Class or the Condomless Sex/Multiple Partners class to be willing to take a free HIVST. However, the Incarceration and Substance Use class was significantly less willing to *purchase* an HIVST, despite being at potentially high risk for acquiring HIV. Among participants who indicated that they would be willing to purchase an HIVST, there were no significant differences in the *amount* participants were willing to pay across latent classes. This finding is particularly noteworthy given that the overwhelming majority of participants were of low socio-economic status and resided in urban neighbourhoods with high levels of concentrated poverty and high rates of HIV infection. Taken together, our findings highlight the need to subsidize HIVSTs, lower HIVST prices, or provide HIVSTs for free, particularly among individuals with a history of incarceration or substance use.

This study adds empirical evidence to recent discussions about uptake and acceptability of HIVST among individuals at high risk for contracting HIV or in communities and neighbourhoods with high rates of infection [23–25]. In 2013, Philadelphia had nearly 700 new HIV diagnoses, adding to the total of nearly 20,000 people living with HIV in the city [30]. Almost 28% of individuals diagnosed in 2011 were concurrently diagnosed with AIDS [31] underscoring the need to find approaches that decrease late entry into life-saving care. Participants in this study overwhelmingly reported they would seek medical care if their HIVSTs were reactive; self-testing may therefore be an important means of reducing concurrent HIV/AIDS diagnoses in urban neighbourhoods with high rates of infection. Our high rates of acceptability also echo the findings of other studies that underscored high levels of acceptability of HIVST in other African American communities, as well as among men who have sex with men [32–34].

In this Philadelphia community and elsewhere, access to HIV screening and treatment are often compounded by other broader social and structural challenges such as the presence of high rates of poverty, joblessness and incarceration. However, our findings suggest that the HIVST is highly acceptable, but that individuals with a history or incarceration or substance use may be less willing to purchase the HIVST. This study provides important information about how best to target HIVST education and outreach to those at highest risk for HIV acquisition, including those with incarceration histories, individuals with a history of substance use, individuals engaging in condomless sex or sex with multiple partners and those in concurrent sexual partnerships. HIVSTs could be disseminated at reduced cost or for free at substance-use treatment centres, upon discharge from prison or jail, and at probation and parole offices.

Our findings also suggest that targeted messaging about HIV risks and HIVSTs should be deployed according to the specific characteristics associated with each latent class. For instance, those in the condomless sex/multiple partnerships group were more likely than others to be young and in between the ages of 13–20; this highlights opportunities to reduce HIV acquisition risks by disseminating HIVSTs and messages about the risks of engaging in condomless sex in collaboration with youth service organizations in urban neighbourhoods. Similarly, those in the concurrent partnerships class were less likely to have a primary care physician or health insurance, highlighting opportunities to promote HIVSTs and messages about the HIV risks associated with having concurrent sexual partnerships in emergency rooms, other places where uninsured individuals might seek care (urgent care facilities), and health insurance linkage programmes.

## Limitations

This study is subject to several limitations. Future replication of the analysis in independent samples would be useful to determine whether the same profiles emerge reliably. When we created the binary willingness to take and buy a home HIV test outcomes, we chose to combine the “neutral” response category with the “unlikely and very unlikely” response categories in order to clearly test hypotheses about willingness to test. By doing so, it is possible that we underestimated willingness to take or buy the test because many participants whose beliefs were neutral may become more willing to take or buy the test in the future. Additionally, we did not administer HIVSTs as part of this study; we were therefore unable to determine whether some of the responses reflected social desirability bias, or whether people with reactive self-test results would seek appropriate medical services or be retained in HIV care. Moreover, respondents in this study had already agreed to undergo testing as part of our large-scale screening and care programme; these participants may therefore not necessarily be representative of the broader community where this study was conducted, some of whom may have self-selected to not participate in this study.

## Conclusions

Our findings highlight important public health opportunities for expanding access to HIVSTs among individuals at high risk for contracting HIV in a community with high rates of infection and limited access to HIV screening and care services. The LCA method also aided in identifying, in an era of scarce resources, individuals at high risk who are willing to take HIVSTs but who may be unable or unwilling to pay the market rate for an HIVST. The results of this study also provided useful information that could inform dissemination of HIVST efforts that are tailored to the characteristics of each specific latent class, particularly among individuals with incarceration histories and those reporting condomless sex with multiple partners. Future programmes should explore how best to deliver and reduce costs of HIVST technology in medically underserved communities and among populations at highest risk for contracting HIV.
